# Administration of Nitrous Oxide Gas

**Published:** 1868-11

**Authors:** Geo. W. Massamore


### ARTICLE III.

### *Administration of Nitrous Oxide Gas.*

By Geo. W. Massamore, D.D.S.

I do not propose to discuss the merits of this invaluable anæsthetic agent, (Nitrous Oxide Gas,) as I trust all have become cognizant by this time, (Dr. Richardson included) of the priceless value of the Gas, to the Dental profession.

I propose to make a few suggestions in regard to administering gas, and my remarks will be directed more particularly to the use of the cork, which is so universally used to prevent the jaws from closing. When I first commenced giving gas, I used the cork, but soon found it to be a nuisance and abandoned its use entirely. My reasons for not using it are: First, it is a great incumbrance to the patients. Second, it prevents free and easy inhalation of the gas, and third, it is greatly in the way of rapid and successful operating. The transitory effect of nitrous oxide gas, devolves on us the necessity of operating with all the dispatch possible, which the use of the cork greatly frustrates. In conclusion I will state how I prevent my patients from closing their jaws. I instruct them not to close their teeth on the pipe through which the gas is inhaled, but to take the pipe into their mouths loosely, simply enclosing it with their lips, and not leave their teeth come in contact with the pipe at all. The result is always the most gratifying, for in no single instance have I yet had any trouble on account of their closing the jaws to prevent my operating successfully, when I operated as above described.

I will also state that I have been administering nitrous oxide gas for nearly two years, and in all that time have not had a single case in which there were any unpleasant effects manifested, but on the contrary, the patients have always been lively and cheerful when the effects of the gas passed off, and expressed the desire of taking it again when they are so unfortunate as to be under the necessity of losing one of their teeth.

---

